# Breast abnormalities in adolescents receiving antiretroviral therapy

**DOI:** 10.4102/sajhivmed.v20i1.1017

**Published:** 2019-11-06

**Authors:** Jackie L. Dunlop, Wiedaad Slemming, Kathryn Schnippel, Caroline Makura, Leon J. Levin, Sarah Rayne, Marnie Vujovic, Cynthia Firnhaber

**Affiliations:** 1 Right to Care, Johannesburg, South Africa; 2 Division of Child Health, School of Paediatrics, University of the Witwatersrand, Johannesburg, South Africa; 3 Health Economics Unit, School of Public Health and Family Medicine, University of Cape Town, Cape Town, South Africa; 4 Department of Surgery, University of the Witwatersrand, Johannesburg, South Africa; 5 School of Clinical Medicine, University of the Witwatersrand, Johannesburg, South Africa; 6 School of Internal Medicine, University of Colorado, Aurora, United States

**Keywords:** adolescent, HIV, antiretroviral, gynaecomastia, breast

## Abstract

**Background:**

Antiretrovirals, particularly efavirenz (EFV), have been shown to cause breast abnormalities in adults. Little is known about the prevalence of these adverse effects among adolescents receiving antiretroviral therapy (ART).

**Objectives:**

The aim of this article was to examine the extent of breast abnormalities in adolescents receiving ART and determine any clinical associations.

**Methods:**

A retrospective record review describing breast conditions in adolescents receiving ART at three facilities in Johannesburg was conducted. Patients aged 10–19 years, who presented from January to December 2014, were included in the study. Analyses were conducted to determine whether EFV was associated with increased breast conditions.

**Results:**

Of the 631 patient records reviewed, 37 (6%) had an abnormal breast event documented; with 24/37 (65%) being male patients. Patients with abnormal breast conditions were 1.5 years older than patients with normal breast development (*p* < 0.0005). Forty-one abnormal breast events were observed in 37 patients, with 20 described as gynaecomastia or lipomastia (49%). Of the 37 patients, 44% (*n* = 19) had concurrent generalised lipodystrophy. Of those with an abnormal breast event, 71% of patients had CD4 counts > 500 cells/µL and were virologically suppressed (*n* = 29). Those on EFV had a significantly higher prevalence of breast abnormalities compared to other regimens (*p* = 0.016).

**Conclusion:**

Of the studied patients, 6% had an abnormal breast condition. The use of EFV and increased age were associated with breast abnormalities in this population. Further research is needed to better understand the implications of this potential side effect.

## Background

The maturation of the HIV epidemic, including increased access to antiretroviral therapy (ART) over the last 20 years, means that increasing numbers of HIV-infected children are entering into adolescence. Growing numbers of adolescents on ART, both boys and girls, are reporting abnormalities of their developing, or already developed, breasts.^1^

During normal breast development, adolescent girls may develop an array of breast abnormalities, including breast hyperplasia or hypertrophy, breast pain, nipple discharge and masses.^2^ Juvenile breast hyperplasia is defined as the uncontrolled overgrowth of breast tissue that occurs in adolescent girls whose breasts develop normally during puberty, but fail to stop growing at the appropriate time.^2^ Quantifying the problem presents difficulties, as there is a spectrum of changes that may be observed. Breast development in boys may be physiological; however, this must be differentiated from gynaecomastia with a pathological origin. The incidence of physiological gynaecomastia ranges from 4% to 69% in adolescent boys who may have palpable breast tissue during puberty.^3^

Antiretrovirals (ARV), particularly efavirenz (EFV), have been shown to cause breast abnormalities in adults.^4,5,6^ Gynaecomastia in adult men and breast hyperplasia or hypertrophy in adult women receiving EFV have been described; however, the same condition is not well explored in adolescents. In the UK and Ireland’s National Collaborative HIV Paediatric Study cohort, 3% of adolescents experienced gynaecomastia (*n* = 56/1873) and, of these, 10 patients presented with severe gynaecomastia, all of whom had current or previous exposure to stavudine (D4T), didanosine and/or EFV. Six patients switched ART regimens and all resolved within 2 years.^1^ In South Africa, a case study described a prepubertal girl who developed pseudogynaecomastia 4 months after initiation onto EFV-based ART, with full resolution 6 weeks following cessation of EFV.^7^ In another case, a 15-year-old HIV-infected boy developed severe, bilateral breasts 2 years after changing to an EFV-containing regimen.^8^

The relationship between puberty and ART appears to affect the characteristics of abnormal breast development and has implications on the short-term management of the breast abnormality and future long-term holistic management of the patient’s HIV care. Therefore, the aim of this study was to establish the frequency and extent of breast abnormalities in adolescents receiving ART in South Africa and to determine whether there were associated ART drugs and comorbidities as well as the investigations and prescribed interventions used in this setting.

## Methods

### Record review and data extraction

A retrospective review of routinely collected medical records was performed at three public health facilities managing adolescent HIV patients in Johannesburg, South Africa. In order for patients’ records to be eligible for review, they had to be aged 10–19 years on ART and had to have presented to their health facility during the study period, that is, 01 January to 31 December 2014. The outcome of interest was whether an adolescent patient on ART had an abnormal breast event. An abnormal event was defined in men as any recorded breast development and in women as breast development that was considered by the clinician, patient or caregiver to be abnormal.

For eligible files, patient demographic profiles and current ART regimen during the study period were recorded, if this information was present in the file. Patient records were screened for breast-related events and for those identified, a comprehensive history of clinical follow-up and ART regimens at the time of the event, as well as details of the actual breast condition were recorded. The description provided by the clinician was captured and used to classify the breast condition. Body mass index (BMI) was calculated using weight (in kilograms) divided by height (in metres squared)^2^. The result was plotted against data sourced from World Health Organization growth charts. Body mass index (kg/m^2^) was plotted according to standard deviation per age (years) for male and female adolescents separately.

Data were collected and managed using the Research Electronic Data Capture (REDCap) tool hosted by the University of the Witwatersrand.^9^ Research Electronic Data Capture is a secure, web-based application designed to support data capture for research studies, providing (1) an intuitive interface for validated data entry, (2) audit trails for tracking data manipulation and export procedures, (3) automated export procedures for seamless data downloads to common statistical packages and (4) procedures for importing data from external sources.^9^

### Data analysis

All statistical analysis was performed by using STATA version 14 (Stata Corporation, College Station, TX, USA).^10^ The prevalence of breast abnormality in this population of HIV-infected adolescents on ART was determined.

Statistically significant associations between the extracted patient characteristics and abnormal breast conditions were tested using Fisher’s exact test for difference in proportions and the Student’s *t*-test for difference of means. A probability value of *p <* 0.05 was designated for statistical significance. Fisher’s exact test was chosen as the appropriate significance test as we expected values less than 9, making a Pearson’s chi-quared test not optimal.

### Ethical consideration

Ethical clearance to conduct the study was obtained from the Human Research Ethics Committee (Medical) University of the Witwatersrand (Ethical Clearance Number: M141134).

## Results

### Patient characteristics

Over the three study sites, 45.9% (631/1376) of files of adolescent patients were eligible for inclusion in the study. The ART clinic characteristics of included records are shown in Table 1.

**TABLE 1:** Antiretroviral therapy clinic characteristics (*n* = 631).

Characteristic	Abnormal breast development *N* = 37	Normal or no breast development *N* = 594
Age at most recent visit (years)†	15.7 (14.3, 16.9)	13.8 (11.8, 16.0)
Age at ART initiation (years)†,‡	8.3 (5.3, 11.0)	8.5 (5.3, 11.5)
Duration of ART (years) †,§	7.5 (4.4, 10.3)	5.6 (2.7, 8.3)

ART, Antiretroviral therapy.

† , Median (IQR).

‡ , 616/631 (97.6%) with recorded age at ART initiation.

§ , 625/631 (99.2%) with recorded ART initiation dates.

### Prevalence of breast abnormalities

The number of files that reported breast abnormalities was 5.9% (37/631). Some patients had breast mentioned multiple times in the file with 41 different abnormal breast events being recorded for 37 patients.

### Demographic characteristics for abnormal breast events

Table 2 shows the patient and breast characteristics for those files in which abnormal breast events were recorded. Abnormal breast conditions were reported at a median of 13.5 years (interquartile range [IQR]: 12.1–15.6 years). All patients with recorded abnormal breast conditions were on ART at the time of the reported event, for a median duration of 4.9 years (IQR: 1.8–6.6) since initiation of ART and 2.0 years since starting their current ART regimen (IQR: 1.2–4.6 years).

**TABLE 2:** Demographic and clinical characteristics of patients with abnormal breast events (*n* = 41).

Characteristic	Description	Count	%
Age at breast event	< 10 years	2	4.9
	10–15 years	28	68.3
	> 15 years	11	26.8
Years on ART from initiation to breast event	< 1	3	7.3
	1	8	19.5
	2	2	4.9
	3	3	7.3
	≥ 4	25	61.0
Years on regimen leading to breast event	< 1	7	17.1
	1	12	29.3
	2	6	14.6
	3	5	12.2
	≥ 4	11	26.8
CD4 count at breast event (cells/µL) †	< 200	4	9.8
	200–500	8	19.5
	500–1000	21	51.2
	> 1000	8	19.5
HIV-1 viral load at breast event (copies/mL) †	Undetectable (< 50)	29	70.7
	Viral load 50–1000	10	24.4
	Viral load > 1000	2	4.9
Age-related BMI (kg/m^2^)	Underweight (< −2 s.d.)	12	29.2
	Normal (−2 to 1 s.d.)	21	51.2
	Overweight (> 1 s.d.)	8	19.5
Description of breast‡	Enlargement	18	43.9
	Breast buds	2	4.9
	Gynaecomastia	12	29.2
	Lipomastia	4	9.8
	Gynaecomastia or Lipomastia	3	7.3
	Breast lump	1	2.4
	None recorded	1	2.4
Severity of breast condition§	Early and small	2	4.9
	Moderate or mild	11	26.8
	Severe or very large	3	7.3
	None recorded	25	61.0
Unilateral or bilateral	Unilateral	5	12.2
	Bilateral	8	19.5
	None recorded	28	68.3

ART, Antiretroviral therapy; BMI, body mass index; s.d., standard deviation.

† , Defined as test taken ± 3 months from the visit date when breast event is recorded.

‡ , The description provided by the clinician was used to classify the breast condition.

§ , Only single count for each event was taken; in case of multiple description of size from clinician, the largest size is taken.

Over two-thirds of patients had CD4 counts higher than 500 cells/µL (70.7%, *n* = 29) and were virologically suppressed as defined by a viral load of 50 copies/mL or fewer (70.7%, *n* = 29). The median CD4 count and viral load in this review were 708.0 cells/µL (IQR: 439.0–957.0 cells/µL) and 176.5 copies/mL (91.5–373.5 copies/mL) respectively.

### Clinical and breast characteristics for abnormal breast events

Table 2 shows that there was a lack of information recorded about the pubertal stage of the adolescents with only four patients (9.8%) with abnormal breast conditions having Tanner staging mentioned in their file.^11^

**FIGURE 1:**
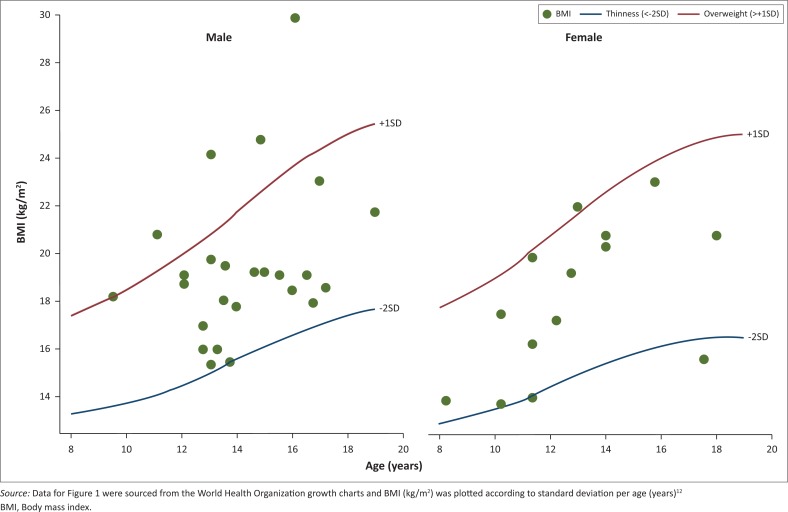
Age-related body mass index (kg/m^2^) scatter plot for breast events reported in male and female adolescents on antiretroviral therapy (male: *n* = 27; female: *n* = 14).

Figure 1 shows a BMI scatter plot for patients at each abnormal breast event. Approximately 12% (*n* = 5/41) were reported as being overweight, which was defined as a BMI plotting one standard deviation above the mean, while approximately 5% (*n* = 2/41) were classified as thin, defined as plotting two standard deviations below the mean. The other 83% had a normal BMI (*n* = 34/41).

### Association of breast abnormalities to patient characteristics and prescribed antiretroviral therapy

Table 3 shows the characteristics of those patient files that had documented abnormal breast events compared to files that had normal breast development or no mention of breast conditions. Patient age, at the latest visit in the study period, with breast abnormalities was significantly older than the group with normal breasts (*p* < 0.0005). Furthermore, a significantly higher percentage of boys (7.9%) than girls (4.0%) had abnormal breast conditions (*p* = 0.043).

**TABLE 3:** Association of breast abnormalities to patient characteristics and prescribed antiretroviral therapy (*n* = 631).

Characteristics	Categories	Patients with abnormal breast (*n* = 37)		Patients with normal breast (*n* = 594)		*p*
		*n*	%	*n*	%	
Age (years)†	During follow-up	15.5	2.0	14.0	2.6	< 0.0005*
	At ART initiation	8.3	4.2	8.5	4.3	0.771
Clinic	Clinic A	19	51.4	321	54.1	0.622
	Clinic B	8	21.6	94	15.8	-
	Clinic C	10	27.0	179	30.1	-
Sex	Female	13	35.1	309	52.0	0.043*
	Male	24	64.9	280	47.1	-
ART‡,§, NRTI¶	Abacavir	28	75.7	347	67.1	0.363
	Zidovudine	1	2.7	20	3.9	0.998
	Stavudine	2	5.4	8	1.2	0.139
	TDF††	2	5.4	49	9.5	0.563
	Abacavir and zidovudine	0	0.0	19	3.7	0.630
	Other NRTI combinations‡‡	2	5.4	8	1.5	0.139
	Incomplete§	2	5.4	66	12.8	0.296
ART‡: Non-NRTI and/or PI	Efavirenz	34	91.9	384	74.3	0.016*
	Other NNRTI§§	0	0.0	3	0.1	0.998
	Lopinavir or ritonavir	0	0.0	50	9.7	0.039*
	Atazanavir or ritonavir	0	0.0	9	1.7	0.998
	Darunavir or ritonavir	0	0.0	1	0.2	0.998
	Non-NRTI + PI	0	0.0	4	0.8	0.998
	Neither: Lamivudine monotherapy	3	8.1	66	12.8	0.606

ART, Antiretroviral therapy; NRTI, nucleoside reverse transcriptase inhibitor.

* , indicates that *p* is significant.

† , Mean (standard deviation [s.d.]).

‡ , Excluded those records for which regimen was not available (*n* = 77).

§ , Lamivudine (3TC) not included in analysis when it is a part of combination ART regimen.

¶ , Nucleoside reverse transcriptase inhibitor (NNRTI).

†† , Tenofovir Disoproxil Fumarate.

‡‡ , Other NRTI combinations include abacavir and stavudine, abacavir and TDF, abacavir and didanosine, stavudine and didanosine, TDF and zidovudine, abacavir and TDF and zidovudine.

§§ , Other NNRTI includes nevirapine and rilpivirine.

¶¶ , Protease inhibitor (PI).

The different associations of ART drugs prescribed in those with abnormal breasts versus those presumed to be developing normally are displayed in Table 3. In both groups, most patients were receiving abacavir with lamivudine (3TC) as their nucleoside reverse transcriptase inhibitor (NRTI) backbone.

A significantly greater proportion of patients with abnormalities were receiving EFV (*n* = 34/37; 91.2%) compared to those with normal breasts (*n* = 384/594; 64.6%) (*p* = 0.016). Of the remaining three events, two abnormal breast events occurred on lamivudine monotherapy and the third one occurred on another NRTI-only holding regimen. The prevalence in these three cases was 4.3% and was non-significantly lower than the overall prevalence of abnormal breast events in the cohort. For those patients taking lopinavir combined with ritonavir, no breast abnormalities were recorded.

### Association of breast abnormalities to antiretroviral therapy characteristics and management of adverse events

All patients with abnormal breast conditions had received EFV as part of a prior or current regimen. The median time of exposure to EFV was 5.5 years. Comparatively, almost 60% of those with abnormal breast conditions had been exposed to D4T with a median time of exposure of 4.9 years. This is displayed in Table 4.

**TABLE 4:** Antiretroviral therapy exposure and adverse events for abnormal breast (*n* = 41).

Characteristic	Variable	*N*	%
EFV exposure *n* = 37 (100%)	Accumulative time on EFV-based regimen†,‡	5.5 years	3.8–8.5
D4T exposure *n* = 22 (59.5%)	Accumulative time on D4T based regimen‡,§	4.9 years	1.8–7.2
Lipodystrophy syndrome	Total	19	46.3
	Lipoatrophy	6	14.6
	Fat accumulation	9	22.0
	No description	4	9.8
Investigations	Total	3	7.3
	Sonography	1	2.4
	Blood tests	2	4.8
Prescribed interventions	Total	21	51.2
	EFV substitution	13	31.7
	Other ART change	3	7.3
	EFV and other substitution	3	7.3
	Diet and exercise	1	2.4
	Tamoxifen prescribed¶	1	2.4

ART, Antiretroviral therapy; EFV, efavirenz.

† , Measured up until first breast abnormality/change from EFV.

‡ , Median (IQR).

§ , Measured up until change from stavudine (D4T).

¶ , Tamoxifen prescribed but not received (taken from the clinical notes).

From Table 4, we see the prevalence of lipodystrophy in the sample investigations and interventions for patients with abnormal breast conditions. Co-morbid lipodystrophy was diagnosed in 46.3% of those with abnormal breast conditions (*n* = 19).

The most common intervention was the substitution of EFV for another ARV (*n* = 16, 39.0%). Of the 19 patients who received any ART drug substitution, only three cases of resolution of the breast condition following ART substitution were observed, with all three cases occurring in the EFV to nevirapine group.

## Discussion

This study aimed to determine whether ART drugs, especially EFV, were associated with breast abnormalities in adolescents. We found that the prevalence of breast abnormalities in this cohort of HIV-infected adolescents on ART was 5.9% and that breast abnormalities in adolescents on ART were associated with current EFV use (*p* = 0.016) and older age in adolescence (*p* < 0.0005).

With such large populations of adolescents on ART in South Africa, common side effects, such as breast abnormalities experienced in a specific age group, become evident. A significant difference in age was observed and adolescents with breast abnormalities were older, and thus on ART for longer, than those without breast conditions. Most abnormalities of the breast in this cohort were experienced at a median age of 15.5 years, which is somewhat later than physiological gynaecomastia in boys, peaking earlier between the ages of 13 and 14 years.^3^

Despite there being more girls in the cohort, there were significantly more breast events seen in male patients. Breast abnormalities in males are easier to detect and define because of the ‘all or nothing’ nature of the abnormalities.

The link between ART drugs and breast abnormalities has not been well established. In this cohort, most patients were receiving EFV in combination with abcacavir (ABC) and 3TC (65.2% of the cohort). From this regimen, only EFV was shown to be associated with abnormal breast development. This suggests that in most patients with breast abnormalities who were receiving ABC/3TC/EFV, EFV remains the likely candidate.

### Theories about abnormal breast development

At present, there are four plausible mechanisms by which ARV drugs or HIV itself can cause breast abnormalities. Firstly, immune reconstitution inflammatory syndrome (IRIS) has been described as a possible mechanism through the production of cytokines which increase breast tissue aromatase activity causing increased oestrogen production.^4^ This was an unlikely cause in our cohort, as IRIS usually takes place within 6 months of starting ART,^13^ and most adolescents with abnormal breasts had received ART for a median time of 5 years, with the median duration of their current ART regimen being 2 years.

Secondly, abnormal breast development in adults is frequently attributed to lipodystrophy syndrome.^14^ Lipodystrophy, defined as the presence of lipoatrophy and/or lipohypertrophy, was by far the most common comorbidity seen in patients in this cohort, with nearly half of those with abnormal breasts reported to have lipodystrophy. Lipodystrophy is associated with receiving D4T and zidovudine.^14^ Despite very few patients with breast abnormalities receiving D4T at the time of censure (*n* = 10), D4T was found to be associated with the development of abnormal breasts (*p* = 0.028). The mechanism is most likely through altered fat accumulation as part of lipodystrophy.

Thirdly, hypogonadism, which is a mechanism thought to cause gynaecomastia in adult male patients on ART, was not explored as a cause in this cohort;^15^ however, the imbalance between oestrogen and androgens during adolescence may play a role in adolescent boys.^3^

Lastly, oestrogen receptor activation by ARV drugs, particularly EFV,^5,16^ is a likely cause in this cohort. At the time of reporting of an abnormal breast event, receiving EFV was significantly associated with the development of breast abnormalities. Furthermore, all patients with abnormal breast conditions had received EFV as part of a previous regimen and their exposure was often long-term with the mean time of exposure being 5.5 years. Efavirenz use was associated with the development of abnormal breast conditions in this adolescent population which is supported by other adolescent and adult studies with similar outcomes.^1,5,6,16^

### Breast abnormalities

Nearly half of the cases of breast abnormality were described as enlargement and just less than half were described using the terms ‘gynaecomastia’ and ‘lipomastia’, which appear to be used interchangeably. The prevalence of severe breast abnormalities for the whole population was 0.5%, which was the same as the prevalence reported in the Collaborative HIV Paediatric Study.^1^

Obtaining a definitive diagnosis of gynaecomastia or breast enlargement remains a challenge because of the lack of available diagnostic modalities and expertise. Sonography is suggested to differentiate between lipomastia related to lipodystrophy and true gynaecomastia; however, in practice, this is not definitive.^4^

### Interventions for breast abnormalities

While over half of patients received an intervention (*n* = 21/41), only three cases were reported to resolve. Of these patients, all three received an EFV to nevirapine substitution, whereas no resolution was seen in patients with any other drug substitution, drug prescription or prescribed lifestyle changes. There was no association established between the resolution of abnormal breast events with substitution to nevirapine; however this was most likely because of the small numbers of breast abnormalities and the limited study period.

While tamoxifen, an antioestrogen drug used for treating gynaecomastia,^3,16^ was prescribed for one patient, it was never received. For this reason, tamoxifen remains an unexplored intervention in this cohort.

Most patients’ breast abnormalities were not reported to resolve and very few were referred on to the next level of care. Of the three that were referred, none received a definitive intervention. This could be attributed to a lack of knowledge on how to manage these cases at a specialist level and thus our findings support that further research is needed to develop management algorithms.

### Comorbidities and concomitant medication

One or more comorbidity was observed in almost three-quarters of patients with abnormal breast conditions. Lipodystrophy was noted in just under half of the patients. Only 12% of this cohort were overweight; however, a relationship between gynaecomastia and obesity was not established.^3^

### Limitations

Although a sample size of 631 patients from 1376 files should be sufficient to answer the research question, a small number of cases were identified, resulting in small subgroups (e.g. boys and adolescents using particular ARVs). It is therefore a possible limitation that the sample size was too small for subgroup comparisons.
There are no comparative studies establishing the frequency of breast abnormalities in this population with a control group (i.e. adolescents who are not HIV-infected or HIV-infected adolescents not on ART).
The prevalence of breast abnormalities in patients less than 10 years old could not be commented on.
Defining breast abnormalities was difficult in girls who experience a spectrum of changes physiologically and pathologically.
Little investigation took place into the cause of breast abnormalities in this cohort.
Adherence to a prescribed treatment is difficult to assess, as it is a subjective measure; however, the effects of ART adherence, which includes HIV-1 viral load results, may be used to estimate adherence.
This record review was performed at three facilities among which the treating clinicians have substantial interaction and, therefore, may have similar algorithms for managing their patients. This may have affected the detection of cases, descriptions used, paucity of investigation as well as interventions used, particularly those of drug substitution.

## Conclusion

Children on ART may develop breast abnormalities during adolescence. It is proposed that the drug choice for adolescent ART, particularly those regimens containing EFV, when interacting with the fluctuating hormonal levels during puberty, may account for this unique presentation of breast abnormality. This study demonstrated that the use of EFV and increasing age were associated with breast abnormalities in this population. The role of EFV in the development of breast abnormalities in both male and female adolescents requires further exploration to determine the appropriate investigations to perform and the most effective interventions to employ.
